# A Pragmatic Low-Salt Diet in Patients with CKD

**DOI:** 10.34067/KID.0000000000000182

**Published:** 2023-07-27

**Authors:** Sunil Bhandari

**Affiliations:** Hull University Teaching Hospitals NHS Trust, Nephrology Anlaby Road Hull, East Yorkshire, United Kingdom

**Keywords:** BP, cardiovascular, CKD, clinical nephrology, health status, kidney dysfunction, nutrition, patient-centered care

We have long been fascinated by the importance of salt to maintain homeostasis of BP and volume and electrolyte balance for optimal cell and muscle function. Indeed, it was the main monetary currency in Roman times, with its benefits as a food preservative and finally as a spice adding taste to food, leading the population to become somewhat dependent on salt like a drug. Salt has also been suggested to have mythical properties; for example, Himalayan pink salt has been purported to benefit the kidneys and bladder.

However, there has been a drive to minimize salt consumption because of its direct effect on lowering BP by an average of 4.2 mm Hg after only 2 weeks of sodium reduction, as noted in the systematic review and meta-analysis of 113 randomized controlled trials (RCTs) with over 12,000 patients.^1^ There is however, limited evidence to determine whether lowering salt consumption leads to clinically significant reductions in ESKD, cardiovascular (CV) events, or all-cause mortality.^2^ Theoretically, salt leads to a reduction in endothelial nitric oxide synthase expression, which affects BP and profibrotic signaling pathways by increased levels of TGF *β* expression, and this may cause organ fibrosis, such as in the heart or kidney.

How much salt is actually needed? Various guidelines have suggested a conservative target of 4 g, but is this enough or is it potentially toxic because there seems to be a J effect seen with studies on salt reduction, particularly concerning CV events and mortality. A major practical challenge is the sustainability of an imposed low-salt diet over the long term, and such diets are inferred (rather than measured) through urinary salt excretion, which in itself is confounded by many factors.

In this issue of *Kidney360*, O'Callaghan and colleagues^3^ in their RCT of 193 people examined the effect of a 1-month pragmatic low-cost intervention (OxSalt care bundle), with an emphasis on empowering people with CKD (eGFR >20 ml/min per 1.73 m^2^) to be proactive and engaged to reduce sodium intake on the effect on 24-hour urinary sodium excretion and secondary effects on BP, proteinuria, and eGFR. They reported a 44 mmol improvement in sodium restriction in the intervention group.

Although the article has several notable limitations, such as adherence to the intervention and its intensity, lack of a dietary record on food choices, and the effect of socioeconomic status on food choices, it adds to the literature given the apparent robust reductions in sodium that were found in relation to the modest intensity of the intervention. This offers a pragmatic approach to sodium restriction that is neither time-consuming nor resource-intensive. Interestingly, in addition, they found from longer-term data evidence of change in personal habits because there was a residual reduction of approximately 1 g in sodium at 11 months.

Using urine sodium is a challenge because it can be affected by several factors, such as medications. Although the authors accounted for some potential biases, there was no information on medications during the trial, such as the use of bicarbonate supplements, tablets with high salt content, diuretics, and renin aldosterone system antagonists, which, as the authors mention, are augmented with reduced sodium intake, and hence, the BP will vary.

Over 90% of dietary sodium is excreted in the urine, but the use of a single spot urine collection to estimate 24-hour sodium excretion and the lack of data on 24-hour urine collections limit the reliability of the results. Indeed, emerging literature suggests that single 24-hour urine sodium measurements are unreliable at assessing sodium intake, primarily because sodium is temporarily stored in soft tissues before being released into the circulation.^4^

What does this study tell us? First, sodium restriction is feasible on the basis of the Oxsalt educational model. Second, simply being in a clinical study seems to change behavior with reduced salt intake, but this does not persist, unlike with the active educational intervention. There was a modest effect on BP, but no tangible benefits on secondary kidney outcomes over at least the short term, so longer-term data will be needed given the variations of outcome in the literature.

A recent systematic review and meta-analysis of 33 studies (*n*=101, 077 participants with CKD) found that a low-salt diet produced a 28% reduction in kidney composite outcome events.^5^ However, there was no evidence that it reduced proteinuria, rate of eGFR decline, risk of all-cause mortality, or CV events.

Smith and colleagues found that there was no significant association between sodium and any kidney outcome in 28,879 participants at high cardiovascular risk over a mean of 4.5 years.^6^ Interestingly, a higher potassium level was associated with lower odds of all kidney outcomes except hyperkalemia, adding further confusion to the literature.

Finally, a prospective community-based cohort of 7976 people with normal kidney function, using a 24-hour dietary recall food frequency questionnaire showed that the risk of CKD development was significantly higher in people with hypertension who consumed <2.08 or >4.03 g/d sodium than in those who consumed between 2.93 and 4.03 g/d.^7^ Thus, again emphasizing a potential J effect on kidney outcomes.

The use of this pragmatic dietary intervention from O'Callaghan and colleagues^5^ will perhaps allow the design of a future RCT to address the longer-term effect on patients with CKD to provide a definitive answer from a kidney perspective. However, this must be balanced with the literature on the effects of salt restriction on cardiovascular outcomes which have failed to consistently confirm a benefit^8–10^ and will need to be included in the design of the definitive trial.

For example, the observational analysis of two cohorts (*n*=28,880) included in the The Ongoing Telmisartan Alone and in Combination with Ramipril Global Endpoint Trial and Telmisartan Randomised Assessment Study in ACE Intolerant Subjects With Cardiovascular Disease trials estimated 24-hour urinary sodium and potassium excretion over 56 months and found that a higher baseline sodium excretion (>8 versus 7–8 g/d) was associated with an increased risk of CV death, myocardial infarction, stroke, and hospitalization for chronic heart failure.^8^ In addition, they showed that higher potassium excretion was associated with a reduced risk of stroke for > 3 g/d. Finally, compared with baseline sodium excretion of 4–5.99 g/d, sodium excretion of >7 g/d was associated with an increased risk of all CV events, and a sodium excretion of <3 g/d was associated with an increased risk of CV mortality and hospitalization for chronic heart failure. Again, the sodium-heart failure RCT of 806 patients with heart failure found that despite at 12 months, an approximate decrease in sodium intake of 628 versus 46 mg/d in the low sodium versus usual care groups, respectively, the primary CV composite outcome and mortality were similar.^9^

Translating this more widely, the Prospective Urban Rural Epidemiology study of 95,767 participants, aged 35–70 years from rural areas found that for every 1 g increase in sodium, there was approximately a 2.86 mm Hg increase in BP,^10^ and sodium intake was associated with 34% more stroke events per 1000 years/1 g increase in sodium intake. Again, there was an apparent J-shaped association where people with low sodium had a higher risk of cardiovascular disease. Hence, it would seem that an ideal sodium intake of between 3 and 5 g/d is associated with the lowest risk.

Future trial designed using the Oxsalt model should also include semistructured interviews to add granularity to the patient experience and have relevant clinical end points to prove that intervention leads to tangible benefits and no clinically relevant detriments. It should also include other populations from ethnic minorities and with all stages of CKD because the current trial was overwhelmingly filled with White/English-speaking participants (89%) with an eGFR of >20 ml/min per 1.73 m^2^ to ensure generalizability to future results in all populations.

In summary, this educational delivery for empowering patients to reduce their salt intake has demonstrated tangible biochemical benefits that last longer beyond the intervention and provides a model for the future of significant patient involvement in clinical trials.

**OXSALT CARE BUNDLE**

Their intervention included three main things in the OxSalt care bundle on the basis of three principles to empower people with CKD:

1. to understand the health benefits of reducing salt intake;
2. to understand how to evaluate the salt content of food;
3. to understand how to select or prepare food that is appetizing and low in salt content.

The intervention included an initial digital briefing, information in electronic and written forms, online resources, and real-time electronic reminders.
